# Analysis of genetic differences between psychiatric disorders: exploring pathways and cell types/tissues involved and ability to differentiate the disorders by polygenic scores

**DOI:** 10.1038/s41398-021-01545-x

**Published:** 2021-08-13

**Authors:** Shitao Rao, Liangying Yin, Yong Xiang, Hon-Cheong So

**Affiliations:** 1 School of Biomedical Sciences, The Chinese University of Hong Kong, Shatin, Hong Kong; 2 Department of Bioinformatics, Fujian Key Laboratory of Medical Bioinformatics, School of Medical Technology and Engineering, Fujian Medical University, Fuzhou, China; 3 Key Laboratory of Ministry of Education for Gastrointestinal Cancer, School of Basic Medical Sciences, Fujian Medical University, Fuzhou, China; 4 KIZ-CUHK Joint Laboratory of Bioresources and Molecular Research of Common Diseases, Kunming Institute of Zoology and The Chinese University of Hong Kong, Kunming, China; 5 CUHK Shenzhen Research Institute, Shenzhen, China; 6 Department of Psychiatry, The Chinese University of Hong Kong, Shatin, Hong Kong; 7 Margaret K.L. Cheung Research Centre for Management of Parkinsonism, The Chinese University of Hong Kong, Shatin, Hong Kong; 8 Brain and Mind Institute, The Chinese University of Hong Kong, Shatin, Hong Kong; 9 Hong Kong Branch of the Chinese Academy of Sciences (CAS) Center for Excellence in Animal Evolution and Genetics, The Chinese University of Hong Kong, Shatin, Hong Kong

**Keywords:** Medical genetics, Genomics, Psychiatric disorders, Diagnostic markers

## Abstract

Although displaying genetic correlations, psychiatric disorders are clinically defined as categorical entities as they each have distinguishing clinical features and may involve different treatments. Identifying *differential* genetic variations between these disorders may reveal how the disorders differ biologically and help to guide more personalized treatment. Here we presented a statistical framework and comprehensive analysis to identify genetic markers *differentially* associated with various psychiatric disorders/traits based on GWAS summary statistics, covering 18 psychiatric traits/disorders and 26 comparisons. We also conducted comprehensive analysis to unravel the genes, pathways and SNP functional categories involved, and the cell types and tissues implicated. We also assessed how well one could distinguish between psychiatric disorders by polygenic risk scores (PRS). SNP-based heritabilities (*h*^2^_snp_) were significantly larger than zero for most comparisons. Based on current GWAS data, PRS have mostly modest power to distinguish between psychiatric disorders. For example, we estimated that AUC for distinguishing schizophrenia from major depressive disorder (MDD), bipolar disorder (BPD) from MDD and schizophrenia from BPD were 0.694, 0.602 and 0.618, respectively, while the maximum AUC (based on *h*^2^_snp_) were 0.763, 0.749 and 0.726, respectively. We also uncovered differences in each pair of studied traits in terms of their differences in genetic correlation with comorbid traits. For example, clinically defined MDD appeared to more strongly genetically correlated with other psychiatric disorders and heart disease, when compared to non-clinically defined depression in UK Biobank. Our findings highlight genetic differences between psychiatric disorders and the mechanisms involved. PRS may help differential diagnosis of selected psychiatric disorders in the future with larger GWAS samples.

## Introduction

Psychiatric disorders are common and more than one-third of the population suffer from at least one kind of disorder in their life [1]. Psychiatric disorders also rank among the top in terms of total disability-adjusted life years [2] lost. Recent analyses based on genome-wide association studies (GWASs) have suggested a moderate-to-high genetic correlation between many psychiatric disorders [3, 4]. On the other hand, although displaying strong genetic correlations, these disorders are clinically defined as independent categorical entities as they each have distinguishing clinical symptoms and often require different treatments [5]. Identifying *differential* genetic variations between these disorders may shed light on how the disorders differ biologically and help to guide more personalized treatment in the future. Another potential clinical application is that genetic markers may help differential diagnosis (DDx) of related disorders. For example, a patient who presents with depression for the first episode may actually be having bipolar disorder (BPD). It is often difficult to distinguish the two diagnoses by clinical features alone at the first presentation, but their treatments differ in important ways. If genetic information can help differentiate BPD from unipolar depression, it will enable more appropriate treatments to be given at an earlier stage of illness.

Most genetic studies to date have focussed on identifying shared loci or genetic overlap between psychiatric disorders [6]. An effort to explore genetic architecture differences between BPD and schizophrenia (SCZ) was made by a recent study [7, 8]. They first compared 9252 BPD cases to 7129 SCZ cases but did not find any single-nucleotide polymorphisms (SNPs) reaching genome-wide significance [7]; however, polygenic risk score (PRS) analysis showed that the score significantly differed between SCZ and BPD patients, indicating that differences between the two disorders have a genetic basis. More recently, they conducted an association analysis with a larger sample size (23,585 SCZ cases and 15,270 BPD cases) and identified two genome-wide significant SNPs [8]. However, the above analyses require individual genotyping data, which might be difficult to access due to privacy concerns. In addition, many of the largest GWAS analyses were conducted by meta-analyses and typically only summary statistics are available.

**Fig. 1:**
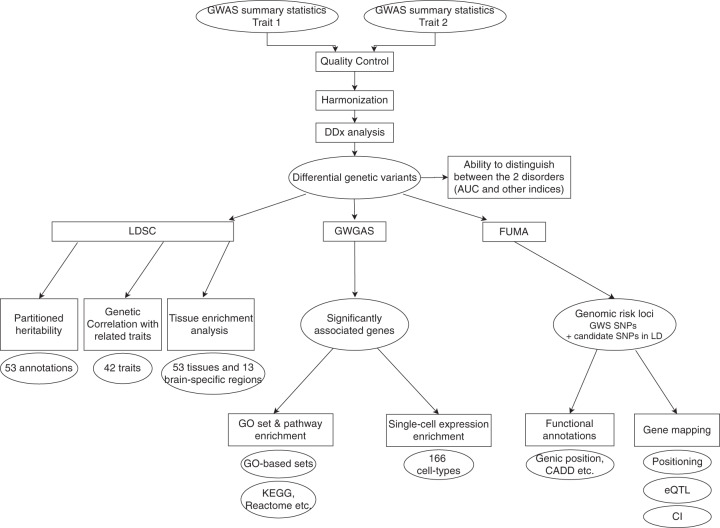
Summary of our analysis framework. GWAS summary statistics of the two traits under study were harmonized and differential genetic associations were identified by the method we described in main text. The power of polygenic scores (derived from the above association test treating the first disorder as ‘case’ and the second one as ‘control’) to differentiate the two disorders was computed. We computed two sets of discriminatory power estimates, one based on *existing* GWAS data, the other based on SNP-based heritability, reflecting the maximum achievable discriminatory power. We also investigated the genetic correlation (mainly using LD score regression [LDSC]) with other possible comorbid traits/disorders. We also performed genome-wide gene-based association study (GWGAS) to identify associated genes and the most relevant tissues, cell types and pathways implicated. As a parallel analysis, we performed functional annotations and mapped the SNPs to relevant genes based on gene positions, expression quantitative trait loci (eQTL) and chromatin interaction (CI) data.

Here we presented a statistical framework and comprehensive analysis to identify *differential* genetic markers covering 18 psychiatric disorders/traits and 26 comparisons, based on GWAS summary statistics (Fig. 1). The analytic framework was successfully validated by simulation studies before applications. Our results based on GWAS summary data showed almost perfect genetic correlation with those obtained via comparing BPD and SCZ individual genotyping data [8] (*r*_g_ = 1.054, se = 0.025), suggesting that our approach is reliable and resembles results from individual-level data analysis.

Importantly, we also conducted an in-depth analysis to reveal the genes and pathways involved and which cell types and tissues were the most relevant in differentiating the disorders (Fig. 1). We also uncovered differences in each pair of disorders in terms of how they are genetically related to different sets of comorbidities. Another novel contribution is that we assessed how well we could distinguish two psychiatric disorders (e.g. major depressive disorder [MDD] vs BPD) using PRS from existing GWAS data, as well as the maximum discriminating ability from all GWAS-panel variants. This may be clinically relevant in the future given the lack of biomarkers for DDx of psychiatric disorders.

## Methods

For details of methods and samples, please also refer to the Supplementary Text.

### GWAS summary statistics

A set of GWAS summary statistics for 18 psychiatric disorders/traits were included (Table 1), which were obtained from several public databases, for example, the Psychiatric Genomics Consortium (PGC, https://www.med.unc.edu/pgc/), the Complex Trait Genetics lab (CTG lab, https://ctg.cncr.nl/) and the UK Biobank (UKBB; http://www.nealelab.is/uk-biobank). Details of the data sets are given in Table 1 and references therein.

**Table 1:** Summary of GWAS data of 18 psychiatric traits/disorders included in this study.

Traits/disorders	Abbreviation	Source	Data type	Cases	Controls	Total *N*	Prevalence (%)^c^
Major depression disorder [9]	MDD	PGC	Binary	59,851	113,154	173,005	13.0 [54]
Bipolar disorder [8]	BPD	PGC	Binary	20,129	21,524	41,653	2.4 [55]
Schizophrenia [17]	SCZ	Pardiñas et al. [17]	Binary	40,675	64,643	105,318	0.5 [29]
Autism spectrum disorder [56]	ASD	iPSYCH&PGC	Binary	18,381	27,969	46,350	2.5 [57]
Attention deficit/hyperactivity disorder [58]	ADHD	iPSYCH&PGC	Binary	19,099	34,194	53,293	6.5 [59]
Post-traumatic stress disorder [52]	PTSD	PGC	Binary	5183	15,547	20,730	3.9 [60]
Anxiety disorder^a^	Anxiety	UKBB	Binary	16,730	101,021	117,751	14.2^d^
Eating disorder [61]	ED	PGC	Binary	3495	10,892	14,477	1.2 [62]
Obsessive-compulsive disorder [63]	OCD	PGC	Binary	2688	7037	9725	2.3 [64]
Insomnia [65]	Insomnia	UKBB&CTG laboratory	Binary	109,389	277,144	386,533	10.0 [66]
Suicide attempts in mental disorder [67]	SA	iPSYCH-PGC	Binary	6024	44,240	50,264	2.7 [68]
Alcohol dependence [69]	Alcohol	PGC	Binary	11,476	23,080	34,556	12.0 [70]
Ever used cannabis [71]	Cannabis	ICC	Binary	43,380	118,702	162,082	4.0 [72]
Psychotic experiences [73]	PE	CNGG-Walters group	Binary	6123	121,843	127,966	5.8 [74]
Neuroticism_Highest_20% [32]	Neuroticism	CTG lab	Binary^b^	78,056	312,222	390,278	20.0^b^
Longest period of depression_Highest_20%	Longest depression	UKBB	Binary^b^	10,133	40,531	50,664	20.0^b^
Probable recurrent major depression (severe)	ProbDep	UKBB	Binary	6304	80,591	86,895	3.55 [75]
Seen doctor (GP) for nerves, anxiety, tension or depression	GPDep	UKBB	Binary	123,528	235,165	358,693	17.3 [54]

*PGC* Psychiatric Genomics Consortium, *UKBB* UK Biobank, *CTG lab* Complex Trait Genetics Lab, *ICC* International Cannabis Consortium, *CNGG* Centre for Neuropsychiatric Genetics and Genomics.

^a^Anxiety disorder: mental health problems ever diagnosed by a professional, including anxiety, nerves, or generalized anxiety disorder.

^b^Continuous neuroticism scores were transformed to binary traits, in which subjects with neuroticism scores within the top 20% were assigned as cases and the others as controls; a similar approach was applied to the period of depression (those within the top 20% were assigned as cases).

^c^The prevalence of traits/disorders refers to estimates of lifetime prevalence based on the cited references.

^d^Prevalence of anxiety disorder is estimated from the UKBB directly.

We included a total of 10 psychiatric disorders in our analysis, including MDD, post-traumatic stress disorder (PTSD), eating disorder (ED), SCZ, BPD, autistic spectrum disorder (ASD), attention deficit/hyperactivity disorder (ADHD), anxiety disorder, obsessive-compulsive disorder (OCD) and alcohol dependence. In principle, we wish to select a wide range of psychiatric disorders covering mood, psychotic, neurotic/stress-related disorders and disorder-related psychoactive substance use. Besides, we also included three other depression-related phenotypes to be compared against MDD from PGC [9]. These three phenotypes were based on the UKBB sample, including longest period of feeling low/depressed, seen doctor (general practitioner (GP)) for nerves, anxiety, tension or depression (to represent self-reported non-specific depression/low mood) and probable recurrent major depression (severe). The latter was derived from several questions based on Smith et al. [10]. In addition to the above, we also included ever used cannabis, insomnia, suicide attempts (SA), neuroticism and psychotic experience (PE) in our analysis as they are closely related to many psychiatric disorders. For details on the choice of phenotypes, please refer to the Supplementary Text.

### Identification of differential genetic markers

We present an analytic approach capable of unravelling the genetic differences between a pair of disorders/traits, relying only on GWAS summary statistics. The method also allows overlap in study samples. In essence, we are ‘mimicking’ a case–control GWAS in which the cases are subjects affected with one disorder and controls affected with the other disorder.

Suppose *T*_1_ and *T*_2_ are two binary traits under study. Let *S* be a biallelic SNP, coded as 0, 1 or 2. For simplicity, we first assume that this is a prospective study of a population-based sample. Based on the principles of logistic regression, we have

2.1 $$\log \left( {\frac{{P(T_1) = 1}}{{P(T_1) = 0}}} \right) = \log \left( {\frac{{p_1}}{{1 - p_1}}} \right) = \beta _{01} + \beta _{11}S + \varepsilon _1$$

2.2 $$\log \left( {\frac{{P(T_2 = 1)}}{{P(T_2 = 0)}}} \right) = \log \left( {\frac{{p_2}}{{1 - p_2}}} \right) = \beta _{02} + \beta _{12}S + \varepsilon _2$$

2.2 $$\log \left( {\frac{{P(T_1 = 1)}}{{P(T_2 = 1)}}} \right) = \log \left( {\frac{{p_3}}{{1 - p_3}}} \right) = \beta _{03} + \beta _{13}S + \varepsilon _3$$

where $p_1 = P(T_1 = 1)$ and $p_2 = P(T_2 = 1)$ denote the probability of the corresponding traits in the collected data set; $\varepsilon _i(i = 1,2,3)$ indicates the error term for corresponding regression model. Based on the definition of odds ratio (OR), for traits $T_1$ and $T_2$, we have:

2.3 $${\rm{OR}}\left( {T_1\,{\rm{vs}}\,{\rm{ctrl}}} \right) = e^{\beta _{11}} = \frac{{\Pr \left( {T_1 = 1|S = s + 1,{\rm{covariates}}} \right)}}{{\Pr \left( {T_1 = 0|S = s + 1,{\rm{covariates}}} \right)}}/\frac{{\Pr \left( {T_1 = 1|S = s + 1,{\rm{covariates}}} \right)}}{{\Pr \left( {T_1 = 0|S = s + 1,{\rm{covariates}}} \right)}}$$

2.4 $${\rm{OR}}\left( {T_2\,{\rm{vs}}\,{\rm{ctrl}}} \right) = e^{\beta _{12}} = \frac{{\Pr \left( {T_2 = 1|S = s + 1,{\rm{covariates}}} \right)}}{{\Pr \left( {T_2 = 0|S = s + 1,{\rm{covariates}}} \right)}}/\frac{{\Pr \left( {T_2 = 1|S = s + 1,{\rm{covariates}}} \right)}}{{\Pr \left( {T_2 = 0|S = s + 1,{\rm{covariates}}} \right)}}$$

Suppose the controls for the two studies come from the same population. In this regard, $\Pr \left( {T_1 = 0|S = s + 1,{\rm{covariates}}} \right)$ and $\Pr \left( {T_1 = 0|S = s,{\rm{covariates}}} \right)$ are approximately the same as $\Pr \left( {T_2 = 0|S = s + 1,{\rm{covariates}}} \right)$ and $\Pr \left( {T_2 = 0|S = s,{\rm{covariates}}} \right)$, respectively. Thus, the OR for differential association between the two diseases can be given as:

2.5 $$\begin{array}{l}{\rm{OR}}\,(T_1\,{\rm{vs}}\,T_2) = e^{\beta _{13}} = \frac{{\Pr \left( {T_1 = 1|S = s + 1,{\rm{covariates}}} \right)}}{{\Pr \left( {T_2 = 1|S = s + 1,{\rm{covariates}}} \right)}}/\frac{{\Pr \left( {T_1 = 1|S = s,{\rm{covariates}}} \right)}}{{\Pr \left( {T_2 = 1|S = s,{\rm{covariates}}} \right)}}\\ \quad \quad \quad \quad \quad \quad \quad \quad \quad \quad \quad \quad \approx \\ \left( {\frac{{\Pr \left( {T_1 = 1|S = s + 1,{\rm{covariates}}} \right)}}{{\Pr \left( {T_1 = 0|S = s + 1,{\rm{covariates}}} \right)}}/\frac{{\Pr \left( {T_1 = 1|S = s,{\rm{covariates}}} \right)}}{{\Pr \left( {T_1 = 1|S = s,{\rm{covariates}}} \right)}}} \right) \div \\ \left( {\frac{{\Pr \left( {T_2 = 1|S = s + 1,{\rm{covariates}}} \right)}}{{\Pr \left( {T_2 = 0|S = s + 1,{\rm{covariates}}} \right)}}/\frac{{\Pr \left( {T_2 = 1|S = s,{\rm{covariates}}} \right)}}{{\Pr \left( {T_2 = 0|S = s,{\rm{covariates}}} \right)}}} \right)\\ \quad \quad \quad \quad \quad \quad \quad \quad \quad \quad \quad \quad \quad \quad \quad \quad \quad \quad \quad = e^{\beta _{11} - \beta _{12}}\end{array}$$

In other words, the effect size of differential association (i.e. trait 1 as case and trait 2 as control) can be derived from the difference of effect sizes of the respective traits. The variance of $\beta _{13}$ can be expressed as:

2.6 $${\rm{Var}}(\beta _{13}) = {\rm{Var}}(\beta _{11} - \beta _{12}) = {\rm{Var}}(\beta _{11}) + {\rm{Var}}(\beta _{12}) - 2{\rm{Cov}}(\beta _{11},\beta _{12})$$

${\rm{Cov}}(\beta _{11},\beta _{12})$ depends on the actual overlap between the samples and the correlation between the two phenotypes. It can be derived from multiplying the square errors (SEs) of the two coefficients with the intercept from cross-trait linkage disequilibrium score regression (LDSC) (see Eq. 6 in [11]). Note that the above derivations only require the regression coefficients (beta), which is the same under a prospective (population-based) or a retrospective design (case–control design where cases may be over- or under-sampled) [12].

Two traits (neuroticism and longest period of feeling depressed/low) were continuous traits. To be consistent with other comparisons which all involves binary traits/disorders, we considered the summary statistics of a corresponding case–control study in which subjects at *top 20%* of the outcome are considered as ‘cases’. The method for deriving binary-trait summary statistics was described in [13]. After computing the differential genetic associations, to further protect against population stratification, we performed genomic control following [14] (i.e. genomic inflation factor was based on LDSC result).

To further check the validity of our approach, we also computed genetic correlation of the results from a GWAS of *BPD* vs *SCZ* from our analytic method against those obtained by comparing the two disorders directly using *individual* genotype data, reported in ref. [8].

### Functional annotations of identified differential genetic markers/gene mapping

The differential genetic variants identified were further explored for their biological functions using FUMA (https://fuma.ctglab.nl/) [15]. SNPs were mapped to genes in FUMA using three different strategies, including mapping by position, expression quantitative trait loci (eQTL) and chromatin interactions (CIs). Details are given in The Supplementary Text.

### Genome-wide gene-based association study (GWGAS) and tissue/cell-type enrichment analysis

*p* Values from SNP-based analysis were utilized for GWGAS analysis in MAGMA [16]. The biological functions of GWGAS-significant genes were further investigated via tissue and cell-type expression enrichment analysis using MAGMA [16] and LDSC [14].

### SNP-based heritability and genetic correlation with related traits

A number of previous studies have shown that common genetic variants as a whole contribute significantly to the susceptibility of individual psychiatric disorders, such as SCZ and major depression [17–19]. Building on previous studies, here we ask a slightly different question. We wish to know whether (and to what extent) common variants as a whole would contribute to the *difference* in susceptibility to different psychiatric disorders. Intuitively, for instance, both SCZ and BPD are highly heritable; however; to what extent do common variants determine why someone may develop SCZ instead of BPD (or vice versa)?

To answer the above question, SNP-based heritability (*h*^2^_snp_) of differential genetic associations was estimated by LDSC and SumHer [20]. We also conducted ‘partitioned heritability’ analysis to identify enriched functional categories [21]. Heritability explained is connected to the predictive power of variants [22]. In this regard, we also estimated the *maximum* ability to differentiate the disorders that can be achieved if all variants on the GWAS panel are accounted for. We followed [22] to compute different predictive indices and graphs. Briefly, we computed the area under the receiver operating characteristic (ROC) curve (AUC), proportion of cases explained by those at the top *k*% of predicted risk, variance of predicted risk and the absolute risk at different percentiles. The graphs that were used to visualize predictive performance included the ROC curve, predictiveness curve and the probability and cumulative density function of predicted risks. This analysis was performed on selected psychiatric disorders and clinical symptoms (PE) for which DDx is considered more clinically relevant.

Genetic correlations (*r*_g_) between the differential genetic variations and 42 potentially related phenotypes were calculated using LDSC (http://ldsc.broadinstitute.org/centers/) [23]. Generally, *r*_g_ reflects how much the non-shared or unique genetic component of the first disorder is genetically correlated with a specific trait, when compared to the second disorder in the pair.

### Potential ability of PRSs from existing GWAS data to differentiate disorders

We performed another analysis to evaluate the ability of PRSs from *existing* GWAS data to distinguish psychiatric disorders. The PRS was based on a case–control study of the corresponding disorders (disorder A as ‘case’ and disorder B as ‘control’). Note that, unlike above, we are *not* focussing on the *maximum* predictive power achievable from all common variants.

An empirical Bayes approach has been proposed to recover the underlying effect sizes and could be used to forecast predictive ability of PRS, based on summary statistics alone [24]. The method has been verified in simulations and real-data applications [24]. Eighteen subsets of genetic variants based on a series of *p* value thresholds (10^−5^, 10^−4^, 5 × 10^−4^, 10^−3^, 5 × 10^−3^, 0.01, 0.03, 0.05, 0.1, 0.2, 0.3, 0.4, 0.5, 0.6, 0.7, 0.8, 0.9 and 1) were used to construct PRS. Note that here we only employed summary statistics to estimate the potential discriminatory ability of PRS; no individual-level genotype data were used.

### Simulation

To verify the validity of our proposed method, we simulated different sets of genotype–phenotype data assuming 300 biallelic SNPs(*N*_snp_ = 300) and 2 disorders. Since the proposed framework is a SNP-based analysis, the number of simulated SNPs will not affect the validity of our simulation. Allele frequency for each simulated SNP was randomly generated from a uniform distribution within [0.05, 0.95]. The number of subjects with each disorder (ncases) was set to [10,000; 20,000; 50,000; 100,000] with a disease prevalence (*K*) of 10%. Here ncases denotes the expected number of cases in the whole simulated population cohort. Given the disease prevalence, the whole simulated population cohort (ntotal) has a sample size of ${\rm{ntotal}} = \frac{{{\rm{ncases}}}}{K}$. The total SNP-based heritability (*h*^2^_snp_) for each trait was set at 0.2–0.4, distributed across all SNPs.

From the simulated population cohort, we simulated two case–control studies with traits A and B as the outcome, respectively. The objective is to simulate GWAS summary statistics that are used as input for our methodology. Suppose the number of cases for traits A and B in the simulated population cohorts are, respectively, $N_A$ and $N_B$, and $N = {\rm{max}}(N_A,N_B)$. To construct the GWAS summary statistics, for trait A, we picked $N_A$ cases and $2N - N_A$ controls from the population. For trait B, we picked $N_B$ cases and $2N - N_B$ controls from the population. We conducted two sets of analysis. In the first set, we only considered cases without comorbid disorders, i.e. all cases identified as having trait A but not trait B were selected as cases ($N_{A\_{\rm{only}}}$) and compared to population controls. Similarly, we conducted GWAS on $N_{B\_{\rm{only}}}$ cases against population controls. This will mimic the situation for disorders that are not diagnosed together like MDD/BPD or SCZ/BPD or if the studies have excluded comorbid patients. In the second set of analysis, we allowed comorbidities for cases, i.e. a proportion of patients with disorder A may also have disorder B (and vice versa). We then recorded the summary statistics from the case–control studies with traits A and B against population controls respectively.

For comparison, we also simulated a ‘real’ GWAS comparing the two disorders. More specifically, we considered patients affected with only one disorder but not the other ($N_{A\_{\rm{only}}}$ and $N_{B\_{\rm{only}}}$). The GWAS comparing $N_{A\_{\rm{only}}}$ and $N_{B\_{\rm{only}}}$ was regarded as the ‘real’ GWAS in our study. To demonstrate the validity of our current method under sample overlap, we also simulated case–control samples with different overlap rates (*P*). Here, *P* indicates the proportion of overlapped samples among all samples selected for each case–control study, i.e., $P = N_{{\rm{ctrl.pverlap}}}/2N$. To adjust the overlap rate, we adjust the number of common controls for both traits (as in practice, the overlap more often occurs in controls).

### Comparing to genes identified from original GWAS of the two disorders

Another straightforward approach for finding genes differentially associated with two disorders is to compare the list of significant genes from the original GWAS of trait A vs controls against those from trait B vs controls. To evaluate whether we will uncover the same set of genes or some unique genes may be found by our proposed framework, we carried out analysis on several disorder pairs (MDD vs BPD, SCZ vs BPD, ADHD vs ASD). We compared the set of differentially associated genes at a false discovery rate (FDR) cut-off of 0.01 using each of the above approaches.

It should be noted that our proposed statistical framework is different and advantageous in several other aspects, when compared to a ‘qualitative’ approach of contrasting the genes/variants found in the two original GWAS. First, our approach can provide a formal assessment of the statistical significance or ‘confidence’ of the differential genes/variants identified. In contrast, to compare the ‘significant’ genes from the two original GWAS, one needs to set an arbitrary cut-off for the inclusion of genes. Second, the proposed method can give an effect size estimate (of the case–control study of trait A vs trait B) of each SNP, which can be further used for downstream analysis like genetic correlations, polygenic scores, transcriptome-wide association studies [25] and so on.

## Results

All Supplementary Tables are also available at https://drive.google.com/file/d/1e0-D7XMNw6-2xCyTqDYQ94bTvUc4V-y3/view or 10.6084/m9.figshare.14850531.

### Simulation results

Table 2 demonstrates our simulation results (please also refer to Table S1). The correlations between the estimated and actual coefficients for the GWAS analysis were very high with different sample sizes of cases. The correlation and root mean square error (RMSE) improved with increased sample size and overlap rate (Table 2). Since the sample sizes for current GWAS summary data are usually >10,000, our proposed method should be sufficiently good to approximate the coefficients from GWAS summary data of corresponding traits. As expected, power increases with larger case sizes and heritability explained by SNPs. In addition, there was no observed inflated type I error at a *p* value threshold of 0.05.

**Table 2:** Simulation results comparing analyses of individual-level genotype data and our presented analytic approach.

Overlap rate	No. of cases	*h*^2^_A_	*h*^2^_B_	Correlation		RMSE		Inferred		Real GWAS	
				Beta	SE	Beta	SE	Power	Type I error	Power	Type I error
0.15	10,000	0.2	0.3	0.98769	0.99789	0.02194	0.00803	0.633	0.040	0.723	0.043
	20,000	0.2	0.3	0.99335	0.99807	0.01634	0.00564	0.740	0.040	0.770	0.037
	50,000	0.2	0.3	0.99766	0.99811	0.00939	0.00359	0.823	0.023	0.873	0.047
	100,000	0.2	0.3	0.99861	0.99811	0.00724	0.00253	0.877	0.047	0.903	0.047
	10,000	0.22	0.32	0.98766	0.99776	0.02253	0.00800	0.653	—	0.723	—
	20,000	0.22	0.32	0.99376	0.99794	0.01618	0.00565	0.723	—	0.787	—
	50,000	0.22	0.32	0.99784	0.99792	0.00941	0.00360	0.833	—	0.880	—
	100,000	0.22	0.32	0.99873	0.99796	0.00724	0.00254	0.877	—	0.910	—
0.25	10,000	0.2	0.3	0.99022	0.99768	0.02077	0.00606	0.660	0.040	0.717	0.043
	20,000	0.2	0.3	0.99645	0.99764	0.01243	0.00437	0.737	0.037	0.770	0.030
	50,000	0.2	0.3	0.99816	0.99785	0.00898	0.00275	0.817	0.043	0.857	0.050
	100,000	0.2	0.3	0.99913	0.99777	0.00608	0.00194	0.870	0.040	0.900	0.027
	10,000	0.22	0.32	0.99144	0.99730	0.02031	0.00605	0.683	—	0.727	—
	20,000	0.22	0.32	0.99637	0.99754	0.01315	0.00436	0.757	—	0.780	—
	50,000	0.22	0.32	0.99831	0.99771	0.00888	0.00275	0.833	—	0.860	—
	100,000	0.22	0.32	0.99923	0.99760	0.00597	0.00194	0.887	—	0.903	—

No. of cases indicates the number of cases we defined for our simulation scenarios; *h*^2^, heritability explained by SNPs.

*RMSE* root mean square error.

We also performed additional simulations that allowed subjects to be comorbid for both traits (i.e. a proportion of patients with trait A can also have trait B, and vice versa). The comorbid proportion was set at ~15%. The correlations of the coefficients (beta) and SE were still very high (mostly >0.99) with similar levels of RMSE (Table S1). The type I error was controlled at 5% (at *p* < 0.05), while the power was modestly reduced when compared to the simulations under no comorbidities.

### Identification of genetic variants differentiating the psychiatric disorders/traits

For the 18 sets of included GWAS summary statistics, we applied the proposed methods to identify differential genetic variants for 26 pairs of comparisons (Table 3). In principle, we selected traits that are similar in nature or commonly comorbid for comparison (please refer to Supplementary Text for details). SNP-based heritabilities are presented in Table 3.

**Table 3:** Identification of differentially associated genetic variants/genes from correlated psychiatric traits/disorders.

Comparisons	Genetic correlation			Differential association GWAS^a^				
	*rg*	*p* value	Intercept	Sig. SNPs	Genomic risk loci	Sig. genes	LDSC-*h*^2^ (se)	SumHer-*h*^2^ (se)
(1) MDD vs psychiatric disorders/traits								
SCZ	0.3857	3.50E−46	0.0548	2312	37	953	0.183 (0.008)	0.220 (0.085)
BPD	0.3387	1.82E−23	0.0679	42	4	174	0.239 (0.013)	0.297 (0.040)
ED	0.1652	1.29E−02	0.0433	94	2	5	0.258 (0.035)	0.319 (0.067)
ASD	0.4466	6.97E−25	0.1441	76	5	17	0.147 (0.015)	—^c^
ADHD	0.5573	1.33E−50	0.1703	167	5	82	0.198 (0.018)	0.162 (0.090)
Anxiety	0.7851	1.87E−32	0.0341	120	5	106	0.284 (0.019)	0.363 (0.035)
Insomnia	0.4706	1.75E−44	0.004	13	3	40	0.083 (0.006)	0.099 (0.011)
Alcohol	0.5893	4.23E−09	0.0389	3	1	0	0.064 (0.016)	0.088 (0.058)
Cannabis	0.2433	8.61E−09	−0.0009	608	7	24	0.122 (0.009)	0.133 (0.016)
SA	0.5639	8.00E−04	0.0069	0	14^b^	0	0.056 (0.023)	0.117 (0.048)
PTSD	0.6095	7.70E−03	0.0064	0	9^b^	0	0.034 (0.024)	0.010 (0.048)
OCD	0.2272	5.00E−04	0.0103	0	12^b^	1	0.344 (0.050)	0.262 (0.081)
(2) MDD vs depression-related traits (from UKBB)								
Recurrent probable depression	1.1036	7.99E−12	0.0617	177	4	110	0.505 (0.033)	0.613 (0.055)
Seen GP for depression	0.9441	6.84E−287	0.0978	22	3	72	0.087 (0.007)	0.111 (0.012)
Long depression (top 20%)	1.0821	1.21E−06	0.0286	3	3	0	0.020 (0.003)	0.021 (0.006)
(3) Neuroticism vs psychiatric disorders								
MDD	0.7507	6.54E−182	0.0644	65	20	4	0.016 (0.001)	0.021 (0.001)
Anxiety	0.7401	7.24E−49	0.1617	4671	57	1232	0.161 (0.006)	0.259 (0.076)
SCZ	0.2293	1.10E−20	0.0102	1733	1214	1	0.013 (0.001)	0.009 (0.003)
Alcohol	0.3754	7.66E−08	0.008	37	3	40	0.022 (0.004)	0.105 (0.004)
(4) Psychotic experiences vs common disorders								
SCZ	0.2014	1.00E−04	0.009	731	10	239	0.356 (0.022)	0.330 (0.043)
BPD	0.1748	2.36E−02	0.0056	25	2	30	0.398 (0.033)	0.587 (0.083)
MDD	0.4957	1.33E−08	0.0072	0	11^b^	0	0.131 (0.022)	—^c^
(5) Other comparisons								
SCZ vs BPD	0.6903	2.13E−181	0.1403	45	3	144	0.202 (0.015)	0.378 (0.071)
ASD vs ADHD	0.3879	4.89E−17	0.3444	335	7	88	0.400 (0.033)	0.438 (0.063)
Alcohol vs Cannabis	0.1482	5.20E−02	0.022	130	2	0	0.132 (0.019)	0.187 (0.027)
Anxiety vs SA	0.0705	7.31E−01	0.0068	1	1	0	0.072 (0.036)	0.137 (0.065)

^a^*MDD* major depression disorder, *SCZ* schizophrenia, *BPD* bipolar disorder, *ED* eating disorder (anorexia nervosa), *PTSD* post-traumatic stress disorder, *OCD* obsessive-compulsive disorder, *ASD* autism spectrum disorder, *ADHD* attention deficit/hyperactivity disorder, *Cannabis* ever used cannabis, *Long depression (top 20%)* subjects with period of depression within the top 20th percentile were considered as cases, *Seen GP for depression* seen general practitioner (GP) for nerves, anxiety, tension or depression, *SA* suicide attempts; alcohol, alcohol dependence.

^b^Sig. SNPs: SNPs with nominal *p* values <5E−08; Sig. Genes: genes with adjusted *p* value (FDR) <0.05 in GWGAS; LDSC/SumHer-*h*^2^: liability-scale SNP-based heritability calculating by the LDSC and SumHer programs, respectively.

^c^The corresponding genomic risk loci are constructed on lead SNPs with *p value <5E−06*, instead of 5E−8 as for other traits.

^d^SumHer returns estimates that are negative, hence we present the results from LDSC only.

These comparisons may be divided into five groups, including MDD vs other psychiatric disorders/traits, MDD vs depression-related traits, neuroticism vs psychiatric disorders, PEs vs three psychiatric disorders and others. Altogether, we identify a total of 11,410 significantly associated differential genetic variants (*p* < 5E−08) and these variants formed up to 1398 genomic risk loci based on LD blocks (Table 3).

Here we highlight selected findings, primarily focussing on MDD vs other psychiatric disorders/traits. Please refer to the Supplementary Text for more detailed results and discussions of other comparisons.

### MDD against psychiatric disorders/outcomes

In this part, we compared MDD with 12 different psychiatric disorders/outcomes, including SCZ, BPD, ED, ASD, ADHD, anxiety disorder, insomnia, alcohol dependence, ever used cannabis, SA, PTSD and OCD. Totally 69 genomic risk loci were identified from the 12 pairs of comparisons (Table 3). Please refer to Tables S2–S13 for detailed results.

#### MDD against SCZ

Among the 12 pairs of comparisons, comparison of MDD and SCZ generate the largest number of genome-wide significant SNPs [2312 SNPs, Table 3 and sub-Table 1 in Supplementary Table 1 (Table S2.1)] which belong to 37 genomic risk loci (Table S2.2).

The three gene-mapping strategies (positional, eQTL and CI mapping) generated a set of 524 unique genes, 94 of which were implicated by all 3 methods (Table S2.5). Additionally, GWGAS analysis identified 953 significant genes (Tables 3 and S2.6). Taken together, 64 genes were implicated by all 4 strategies. Among them, *CACNA1C* was predicted to have a very high probability of loss-of-function mutation intolerance (pLI score = 1; Table S2.5). Genes differentiating MDD and SCZ were mainly enriched in the cortex, the anterior cingulate cortex (ACC; BA24) and the frontal cortex (BA9) regions (Table S2.8; FDR < 6.0E−04). Cell-type enrichment analysis suggested strong associations with several kinds of neurons in the cortex and prefrontal cortex (Table S2.9). Moreover, this analysis also identified associations with neurons in the midbrain, hippocampus and lateral geniculate nucleus (LGN) regions (Table S2.9). Conditional analyses suggested neurons in the cortex, GABAergic neurons in the midbrain and pyramidal neurons in the hippocampus as *independent* contributing neurons (after controlling for other cell types; Table S2.10).

In gene-set enrichment analysis, the 953 GWGAS significant genes were enriched in a number of biological GO sets, including generation of neurons, regulation of nervous system development and central nervous system neuron differentiation (Table S2.11). We also conducted genetic correlation analysis in which SCZ was defined as the ‘case’ and MDD as (pseudo-)‘controls’. Note that a positive genetic correlation indicates that the ‘case’ disorder is more positively associated with the studied trait genetically than the (pseudo-)‘control’ disorder, and vice versa. For example, we observed inverse genetic correlations (*r*_g_) with insomnia, neuroticism, coronary artery disease (CAD) and mean hippocampal volume, among others. This suggested that MDD has stronger positive genetic correlations with the above traits/disorders compared to SCZ. Findings of this type may not only shed light on different patterns of comorbidities but may also be clinically informative. For instance, the significant inverse *r*_g_ with CAD suggested that, compared to SCZ patients, MDD patients may be more genetically predisposed to CAD.

#### MDD against BPD, ED, ASD, ADHD, anxiety disorder, insomnia, alcohol dependence and cannabis use

In these 8 pairs of comparisons, we identified 32 differential genomic loci (Tables 3 and S3–S10). The comparison between MDD and BPD revealed the largest number of significant genes based on GWGAS (174 genes; Tables 3 and S3.6). Details are presented in Tables S3–S10 and the Supplementary Text.

### MDD against depression-related traits

Here we tried to identify differential genetic variants from three pairs of comparisons between MDD and three depression-related phenotypes (probable recurrent severe depression, seen GP for anxiety/depression and longest period of low/depressed). Ten risk loci were identified (Table 3/Tables S14–S16).

#### MDD against depression defined in UKBB

First, we compared MDD (from PGC; majority clinically defined) against probable recurrent major depression (severe) (ProbDep). We identified four risk loci (Tables 3 and S14.2), including one in the extended MHC(xMHC) region. Gene-based test revealed 110 significant genes. Tissue enrichment analysis highlighted the cerebellar hemisphere, nucleus accumbens and frontal cortex as the most enriched regions. Cell-type enrichment analysis suggested that the significant genes were associated with GABAergic, dopaminergic and other types of neurons in LGN, middle temporal gyrus, hippocampus, midbrain and cortex regions (Table S14.9). Genetic correlation analysis showed that MDD-PGC was more positively genetically correlated with most other psychiatric disorders (e.g. SCZ/BPD/ASD/ADHD) as well as CAD when compared with ProbDep (Table S14.13). We also compared MDD against ‘seen GP for nerves/anxiety/depression’ (GPDep), with detailed results shown in Table S15.

#### MDD against duration of longest period of feeling low/depressed (top quintile as case)

Three genetic risk loci were identified, including the *GRIK2* gene. The gene codes the Glutamate Ionotropic Receptor Kainate Type Subunit 2, suggesting that glutamatergic transmission may be one factor with differential associations between susceptibility to depression and severity (as reflected by duration) of illness.

### Neuroticism against SCZ/MDD/anxiety disorder/alcohol dependence

These comparisons were made based on relatively high association of neuroticism with these disorders [26–28]. We identified 1294 genomic risk loci (Table 3). Please refer to Tables S17–S20 for details.

### PEs against SCZ/BPD/MDD

We identified 10 and 2 genomic risk loci from comparison of PEs against SCZ and BPD, respectively, but not from PEs against MDD (Table 3/Tables S21–S23).

### Other comparisons

We also applied the proposed methods to SCZ against BPD, ADHD against ASD, alcohol dependence against ever used cannabis and anxiety disorder against SA. We identified 3, 7, 2 and 1 genomic risk loci from each of the comparison, respectively (Table 3). Please refer to the Supplementary Text and Tables S24–S27 for details.

### Top differentially associated genes

For the comparisons involving MDD, the top five differentially associated genes from each comparison (based on gene-based analysis using MAGMA) are listed in Table 4. For full results, please also refer to Tables S2–S27.

**Table 4:** Top five differentially associated genes from each comparison based on gene-based analysis using MAGMA (listing comparisons involving MDD).

Comparison	Top 5 differentially associated genes	*p*	FDR-adjusted *p*
SCZ vs MDD	***PPP1R16B***, ***HIST1H4L***, ***DPYD***, ***PITPNM2***, ***NGEF***	<5.44E−12	<1.99E−08
BPD vs MDD	***HAPLN4***, ***TRANK1***, ***VPS9D1***, ***MAD1L1***, ***NDUFA13***	<4.24E−07	<1.33E−03
MDD vs ASD	***MACROD2***, ***XRN2***, ***WDPCP***, ***EGR2***, ***FZD5***	<4.00E−06	<1.27E−02
MDD vs ADHD	***CDH8***, ***MEF2C***, ***KDM4A***, ***PTPRF***, ***KCNH3***	<2.02E−07	<7.48E−04
MDD vs ED	***ERBB3***, ***SUOX***, ***FAM19A2***, ***CRTC3***, ***RAB5B***	<5.58E−06	<2.09E−02
MDD vs anxiety	***BTN3A2***, ***HIST1H2BN***, ***PTPN1***, ***ZKSCAN4***, ***PGBD1***	<3.54E−08	<1.33E−04
MDD vs insomnia	***BTN3A2***, ***HIST1H2BN***, ***ZSCAN9***, ***SYNGAP1***, ***RAB1B***	<4.98E−07	<3.81E−03
MDD vs alcohol	*MTFR1*, *ATF6B*, *KREMEN2*, *SLC25A52*, *ALPK1*	<1.35E−04	<4.47E−01
MDD vs cannabis	***CADM2***, ***C10orf32-ASMT***, ***AS3MT***, ***ACTL8***, ***ARID1B***	<3.80E−07	<1.43E−03
MDD vs SA	*NCL*, *ST8SIA5*, *COA4*, *PDE4B*, *SLBP*	<1.46E−04	<3.99E−01
MDD vs PTSD	*ATP6V1E1*, *MYO5B*, *ZYG11A*, *GNA15*, *UBA3*	<3.04E−04	<8.89E−01
MDD vs OCD	***KIT***, *PLAG1*, *FGF19*, *PPIG*, *TXNL1*	<3.31E−05	<8.88E−02
MDD vs probable recurrent major depression (severe)	***HIST1H2BN***, ***BTN3A2***, ***ZKSCAN4***, ***PGBD1***, ***PTPN1***	<3.89E−08	<1.47E−04
MDD vs seen GP for depression	***PTPN1***, ***BTN3A2***, ***ZKSCAN4***, ***HIST1H2BN***, ***PGBD1***	<1.54E−07	<5.81E−04
Longest depression vs MDD	*FBXW4*, *C11orf42*, *AC079602.1*, *ANAPC11*, *HIST1H2BM*	<1.56E−03	<8.33E−01
Neuroticism vs MDD	***MAPT***, ***WNT3***, ***CRHR1***, ***KANSL1***, *NSF*	<1.73E−05	<6.50E−02
Psychotic experiences vs MDD	*FAM168A*, *SHPRH*, *SPAM1*, *ADRB2*, *POC1B*	<1.48E−04	<3.66E−01

Genes with FDR < 0.05 are in bold. We focus on comparisons involving MDD in this table; for top genes involving other pairs of disorders, please refer to the Supplementary Tables.

### Distinguishing between disorders based on PRS

#### Potential ability to distinguish between disorders based on PRS derived from current GWAS data

In the analysis, we assume that each subject is having either one of the disorders. Taking SCZ vs MDD as an example, we assume the DDx has been narrowed down to either SCZ or MDD. The prior probabilities (without genetic information) of being affected with either disorder are based on lifetime prevalence of the disorders [29, 30]. For example, here we assume a person has ~13%/0.5% = 26 times of being affected by MDD than by SCZ, in the absence of additional information. Our analytic framework actually allows more flexible setting of these prior probabilities, although we made simpler assumptions here. We expect that, with the addition of polygenic scores, one would be able to differentiate the disorders more accurately. A good prediction model leads to more spread-out predicted risks and larger relative risks (RRs) when we compare subjects at the top and bottom percentiles.

Subjects at the lowest 5th percentile of the PRS distribution (SCZ as ‘case’ and MDD as ‘control’) have markedly lower risks of SCZ than that of MDD compared to the population average. In this case, the predicted absolute risks of SCZ and MDD were 0.792 and 99.208%, respectively. The RR of MDD vs SCZ was therefore ~125.3 for a person with PRS at the bottom 5th percentile (average RR = 26 as described above; Table S2.15). With an increase in PRS, the risk of SCZ became higher, while the risk of MDD reduced. Subjects at the highest 5% of the risk score (of SCZ vs MDD) had a substantially decreased RR of 10.16. At the start, we assume ~26 times higher risks of MDD than SCZ based on overall lifetime risks; a reduction to 10.16 times is a relatively large change. We also present the RR of the ‘case’ disorder by comparing individuals at the highest and lowest *x*th percentiles (Table 5). For example, the estimated RR of SCZ was 11.31 if we compare those at the highest 5th against those at the lowest 5th percentile. For SCZ vs BPD and BPD vs MDD, the corresponding RRs (for the 1st disorder) were 3.29 and 2.82, respectively.

**Table 5:** Ability to discriminate psychiatric disorders by polygenic risk scores (PRSs).

Comparison	Discriminating ability			Relative risk of the first disorder comparing the top against the bottom percentiles (based on existing GWAS)			
	Max. based on SNP-based *h*^2^	Polygenic risk scores (based on existing GWAS)		Top 5th vs lowest 5th	Top 10th vs lowest 10th	Top 20th vs lowest 20th	Top 30th vs lowest 30th
	AUC	Best *p*-thres	AUC	Percentile	Percentile	Percentile	Percentile
SCZ vs MDD	0.763	0.005	0.694	11.31	6.61	3.46	2.17
BPD vs MDD	0.749	0.005	0.602	2.82	2.24	1.70	1.39
MDD vs ED	0.780	0.0005	0.587	1.09	1.07	1.04	1.03
MDD vs ASD	0.694	0.01	0.551	1.10	1.08	1.05	1.03
MDD vs ADHD	0.707	0.005	0.583	1.38	1.29	1.18	1.11
MDD vs anxiety	0.744	0.001	0.570	1.54	1.40	1.25	1.15
MDD vs OCD	0.800	0.005	0.566	1.12	1.09	1.06	1.04
SCZ vs Psychotic experiences	0.828	0.005	0.686	9.19	5.62	3.11	2.03
BPD vs Psychotic experiences	0.801	0.005	0.584	2.02	1.73	1.43	1.25
MDD vs Psychotic experiences	0.668	0.00001	0.524	1.09	1.07	1.04	1.03
SCZ vs BPD	0.726	0.005	0.618	3.29	2.53	1.84	1.46
ADHD vs ASD	0.804	0.001	0.622	1.50	1.37	1.23	1.14

*AUC* area under the ROC curve, *SNP-based h*^*2*^ SNP-based heritability based on the entire GWAS panel, *Max* the maximum discriminating ability (in AUC) for the two traits in each comparison based on SNP-based heritability, assuming that all risk variants in the GWAS panel are found. *Best p-thres* the *p* value threshold for PRS analysis leading to the highest AUC. The last four columns show the relative risk of the first disorder at the top *x*th against the lowest *x*th percentiles of PRS (derived from treating disorder A as ‘case’ and disorder B as ‘control’). MDD vs PTSD was not listed above as the results from GWAS analysis were too weak in power such that the AUC based on PRS of current GWAS data cannot be estimated. The maximum AUC (based on SNP-based *h*^2^) was estimated to be 0.588.

For most comparisons, the AUC based on PRS of *existing* GWAS data were modest, with several pairs showing AUC > 0.6 (Table 5). For example, we estimated that AUCs for distinguishing SCZ from MDD, BPD from MDD and SCZ from BPD were 0.694, 0.602 and 0.618, respectively. The AUCs of distinguishing SCZ vs PE and ADHD vs ASD were estimated to be 0.686 and 0.622, respectively.

#### Maximum AUC based on SNP-based heritability (i.e. all GWAS-panel variants)

The maximum AUC attainable (at SNP-based heritability) is presented in Table 5. The levels were much higher than the current AUC, indicating room for improving discriminating ability by increasing sample sizes. For example, based on SNP-based heritability, the AUCs for distinguishing SCZ from MDD, BPD from MDD and SCZ from BPD were 0.763, 0.749 and 0.726, respectively. We also computed other predictive indices/graphs, which are shown in Supplementary Tables.

### Comparing to genes identified from original GWAS of the two disorders

The results are presented in Tables S28–30. Briefly, in the comparison of ASD vs ADHD, 40 genes had FDR < 0.01 based on our analysis, of which 27 did not overlap with the genes found by simple comparison of the original GWAS (at FDR < 0.01). For BPD vs MDD, the corresponding numbers were 38 and 5; for SCZ vs BPD, the corresponding numbers were 49 and 15.

We note that the two approaches are different and the results are not directly comparable, as directly comparing genes found from the two original GWAS does not provide a formal assessment of the statistical significance of individual genes. The results are shown for reference and as an exploratory analysis.

## Discussion

The present study applied a simple yet useful analytic framework to identify differential genetic markers for a board range of psychiatric disorders/traits. We conducted detailed secondary analysis to identify the genes, pathways and cell types/tissues implicated. From the 26 pairs of comparisons, we identified a total of 11,410 significantly associated differential variants, 1398 genomic risk loci and 3362 significant genes from GWGAS with FDR < 0.05.

### SNP-based heritability(*h*^2^_snp_) of differential genetic associations

Here found that the SNP-based heritabilities were significantly different from zero for almost all comparisons between psychiatric disorders, with some having moderately high heritabilities. This suggests that genetic differences (due to common variants) may at least partially underlie the differences in susceptibility between psychiatric disorders, even for closely related ones such as MDD and anxiety disorders.

For MDD and comparisons with other disorders, we observed the highest *h*^2^_snp_ in the comparison with SCZ, BPD, ED and anxiety disorders. For instance, despite substantial symptom overlap [31] between MDD and anxiety disorders, the *h*^2^_snp_ is among the highest at ~36% (by SumHer; on liability scale). On the other hand, *h*^2^_snp_ was estimated at ~1% only when comparing MDD to PTSD. A possible explanation is that environmental factors (e.g. traumatic stressors must be present for PTSD but not for MDD) may play an important role in explaining the differences between the two disorders. For neuroticism against other psychiatric disorders, the *h*^2^_snp_ were in general low; however, *h*^2^_snp_ for neuroticism itself was only ~10% [32].

We wish to highlight a difference between genetic correlation (between two traits) and the *h*^2^_snp_ from the differential association test. Two variables can have a high correlation if there is a strong linear relationship, but the actual values of the variables can differ. It is possible that two traits have a high genetic correlation (*r*_g_), but as the effect sizes of SNPs can differ, *h*^2^_snp_ can still be substantial. There are several caveats when interpreting *h*^2^_snp._ First, large samples are often required for *h*^2^_snp_ analysis. However, for several disorders sample sizes were relatively moderate (e.g. OCD/ED); as such, the estimates could be imprecise. Also, contribution of rare variants and other ‘omic’ changes (e.g. epigenetic changes) were not captured by *h*^2^_snp._ Moreover, estimation of *h*^2^_snp_ is subject to model assumptions [20] of genetic architecture, which can vary across diseases.

### Potential ability of PRS from existing GWAS data to differentiate disorders

A potential translational aspect is to make use of PRS from SNPs to distinguish between psychiatric disorders, which has been raised, for example, by ref. [33] in a recent review. This is particularly relevant in psychiatry due to the lack of objective biomarkers. In an earlier work, Hamshere et al. [34] found that PRS of SCZ was able to differentiate schizoaffective BPD patients from non-schizoaffective BPD patients. In a more recent study, Liebers et al. [35] studied whether PRS may discriminate BPD from MDD. They found that subjects at the top decile of BPD PRS were significantly more likely to have BPD than MDD, when compared to those in the lowest decile. The estimated OR was 3.39 (95% confidence interval 2.19–5.25), which is comparable to our RR estimate (2.24) (see Table 4; RRs are usually smaller than ORs).

Among the comparisons, DDx between MDD and BPD is one of the most clinically relevant. Based on the present GWAS data, the AUC for discriminating BPD vs MDD is 0.602 (at the best *p* value threshold), which is modest. However, PRS may be more informative for individuals at the extreme end of the score. The discriminating power between BPD and SCZ was similarly modest (best AUC = 0.618) but the AUC for SCZ vs MDD was much higher (0.694). Clinically, major depression (mainly psychotic depression) may be a DDx for first-episode psychosis [36]; it may be interesting to study whether PRS can help distinguish SCZ from MDD in such patients. We also estimated the maximum discriminatory ability by PRS based on *h*^2^_snp_ (i.e. assuming all common variants are found); the maximum AUC for MDD vs BPD, SCZ vs BPD and SCZ vs MDD were 0.749, 0.726 and 0.763, respectively. These findings suggest that, with larger GWAS sample sizes, PRS may become more informative and may help DDx. Another interesting analysis is on how well PRS can differentiate ‘PE’ against psychiatric disorders such as SCZ, BPD and MDD, which we found high AUC based on *h*^2^_snp_ but poorer discriminatory power using existing GWAS data (see Supplementary Text).

Several limitations are worth noting. For some comparisons (e.g. MDD vs other disorders), comorbidities are possible. For our PRS-based DDx, as stated before, it was assumed that the subject is having either one of the disorders. For example, we assume the DDx has been narrowed down to either SCZ or MDD in our analysis of SCZ vs MDD. In practice, the above assumption may be true for some disorder pairs (e.g. SCZ vs BPD; BPD vs unipolar depression) but may not hold for others (for which a patient can have both disorders at the same time). As such, the PRS analysis results and AUCs should be viewed with caution, although we believe they are still of scientific interest. On the other hand, in the presence of comorbidities, the PRS approach may still be able to inform whether a person has a higher genetic predisposition to one disorder than the other, say SCZ compared to MDD. Whether this may be of importance clinically will require further studies. For example, an interesting question is that whether a specific treatment (e.g. for disorder *A*) may be more effective in patients with higher genetic predisposition to disorder *A* (or vice versa), even if comorbidity is possible.

In practice, we expect clinical symptoms and features still remain very important in making DDx. Genomic data may provide additional discriminatory power when integrated with clinical features. Also, since we relied on summary statistics, we applied an analytic approach [24] to estimate the AUC from current GWAS samples. Limitations of this methodology were detailed in [24]. Mainly, we assume the predictive model will be applied to the same population as the training data. Nevertheless, as patients with the same psychiatric disorder can be heterogeneous, and PRS may need to be applied across different ethnic groups, the estimated AUCS may be optimistic in this regard. Ideally, predictive power should be further evaluated in an independent set with individual genotype data. In addition, our analytic approach for forecasting AUC assumed a (standard) *p* value thresholding and LD clumping(*P* + *T*) approach. This approach is widely adopted, but newer PRS modelling methodologies (e.g. LDpred; see [37] for a review) may be used to further improve predictive power.

### Comparison of MDD-PGC with depression-related traits in UKBB

We performed another interesting comparison between MDD-PGC [9] and other depression-related traits from UKBB. The former group was mainly composed of clinically diagnosed MDD, while the latter group was largely defined by self-reporting. For example, for recurrent probable major depression (severe) (ProbDep), it included subjects who reported feeling depressed for 1 week, with ≥2 episodes for ≥2 weeks and have visited a psychiatrist (please also refer to Smith et al. [10] and https://biobank.ctsu.ox.ac.uk/crystal/crystal/docs/MentalStatesDerivation.pdf). The other phenotype was having seen GP for depression/nerves/anxiety. Neither trait involved assessment of clinical symptoms as described in Diagnostic and Statistical Manual of Mental Disorders/International Classification of Diseases. Based on our analysis, MDD-PGC appeared to be more strongly genetically correlated with other psychiatric disorders (e.g. SCZ/BPD/anorexia/ASD/ADHD) and other outcomes such as CAD, when compared with non-clinically defined depression in UKBB. Interestingly, while *r*_g_ between MDD-PGC and UKBB depression traits were high, the SNP-based heritability from differential genetic analysis was significantly larger than zero. One possible explanation is that, while many susceptibility genes may be shared between them, the effect sizes may differ. Another point to note is that *r*_g_ based on LDSC may be overestimated in case–control studies due to difficulties in handling covariates [38]. The latter has been reported in [39] when comparing LDSC against a more sophisticated method PCGC [38].

Recently, Cai et al. [39] suggested that depression traits defined by ‘minimal phenotyping’ (ProbDep and GPDep included here also belonged to ‘minimal phenotyping’) are genetically different from strictly defined MDD. For example, they have lower *h*^2^_snp_ and have worse predictive power in MDD cohorts. Cai et al. focussed on comparisons of different definitions of ‘depression’ within UKBB, while here we mainly compared the genetic architecture of MDD-PGC against traits in UKBB; we also employed a different statistical approach. Our results supported differences in genetic basis between different definitions of depression and calls for more in-depth phenotyping to study depression and related traits.

We would like to highlight an important limitation of the above comparisons. Although the MDD-PGC sample mainly comprises clinically diagnosed MDD, several limitations cannot be addressed in this analysis. For example, the proportion of patients with comorbid disorders is unknown. Also, the diagnostic approach, inclusion/exclusion criteria, clinical features and other sample characteristics may differ across substudies.

### Tissue/cell-type enrichment analysis

In view of the large number of comparisons performed, we just highlighted a few results for discussion. The tissue/cell-type enrichment analysis implied that the frontal cortex (BA9) and ACC (BA24) may be implicated in the *difference* between several disorders, such as MDD against SCZ/BPD, neuroticism against MDD/alcohol dependence and PEs against SCZ/BPD. BA9 contributes to the dorsolateral and medial prefrontal cortex, dysfunction of which underlies many cognitive and behavioural disturbances that are associated with psychiatric disorders, such as SCZ, MDD, ADHD and ASD [40, 41]. The ACC is involved in many functional roles of the brain, including affective, cognitive and motor aspects [42]. A number of studies have suggested that functioning alterations in the ACC may be implicated in psychiatric disorders, such as MDD [43], BPD [44] and SCZ [45]. It is possible that different patterns of dysfunctioning in these brain regions may underlie the differences between the disorders.

Cell-type enrichment analysis may also help to pinpoint the cell types (and brain regions) involved in differentiating the disorders. For example, when comparing MDD vs anxiety disorders, the most enriched cell types were GABAergic neurons from hippocampus, midbrain and temporal cortex. Interestingly, benzodiazepines, one of the most widely prescribed drugs for anxiety, acts on the GABAergic pathway, while antidepressants primarily target the monoamine system. With increasing amount of single-cell RNA sequencing data in the future, cell-type enrichment analysis may delineate more precisely the specific type of neurons involved.

### Genetic correlation analysis

We just briefly highlight a few examples of our findings. For example, in the comparison of BPD vs MDD, we observed positive genetic correlation (*r*_g_) with childhood intelligence and level of education (Table S2). This suggests that BPD, when compared to MDD, is more strongly genetically linked to these traits (in the positive direction). This is in line with a previous study which reported that low intelligence was associated with severe depression and SCZ but not with BPD [46]. On the other hand, another study reported that, in men with no psychiatric comorbidity, both low and high intelligence are risk factors for BPD [47]. However, these are epidemiological studies and further studies are required to validate our findings and to reconcile with epidemiological findings. Slightly unexpectedly, we also observed positive *r*_g_ of BPD vs MDD with anorexia nervosa (AN), although AN is more commonly comorbid with depression clinically. Similarly, when comparing SCZ vs MDD, positive *r*_g_ with AN was also observed. This could suggest that MDD in AN is more strongly influenced by environmental factors [48].

Genetic correlation analysis may also shed light on the brain regions implicated. For the comparison of SCZ vs MDD, we observed a significant negative correlation with hippocampal volume, which was corroborated by associations in the hippocampus region in cell-type enrichment analysis. Both SCZ and MDD patients were reported to have smaller hippocampal volumes compared to healthy controls [49, 50]. However, a comparative study showed that there was a larger reduction in hippocampal volumes in SCZ compared to MDD [51]. Our *r*_g_ analysis appeared to support this finding.

### Other limitations

Many limitations of this study have been detailed above. As for other limitations, we note that the methodology assumes the controls of both GWAS data sets originate from a similar population. If the heterogeneity is high (e.g. from different ethnic groups), the estimates may be biased. As a related limitation, most of the studies were based on Europeans; the effects of genetic loci may differ across populations, and PRS derived from Europeans may have poorer predictive abilities in other ethnicities.

Besides, information on possible comorbidities is not available from most GWAS data sets. For example, in a comparison of MDD vs OCD, some OCD patients may have comorbid MDD. To a certain extent, this is similar to the use of unscreened controls in genetic studies, which may lead to reduction of power to detect genetic variants but generally does not increase risks of false positives [52, 53]. Our simulations also supported the validity of the proposed statistical approach in the presence of comorbidities. However, from a clinical perspective, comorbidities also affect the interpretation of PRS-based differentiation of disorders, which has been discussed in detail above.

## Conclusions

Our proposed analytic framework successfully identified a number of differential genomic risk loci from 26 pairs of comparisons of psychiatric traits/disorders. Moreover, further analysis revealed many novel genes, pathways, brain regions and specific cell types implicated in the differences between disorders. We also showed that PRS may help differentiation of some psychiatric disorders to a certain extent, but further clinical studies are required.

## Supplementary information

Supplementary Text

Supplementary Dataset

## Data Availability

R code for the analytic framework to identify differential genetic markers (based on GWAS summary statistics) is available at https://github.com/LiangyingYin/DDx.
