# Correction: Analysis of genetic differences between psychiatric disorders: exploring pathways and cell types/tissues involved and ability to differentiate the disorders by polygenic scores

**DOI:** 10.1038/s41398-022-02012-x

**Published:** 2022-06-07

**Authors:** Shitao Rao, Liangying Yin, Yong Xiang, Hon-Cheong So

**Affiliations:** 1 School of Biomedical Sciences, The Chinese University of Hong Kong, Shatin, Hong Kong; 2 Department of Bioinformatics, Fujian Key Laboratory of Medical Bioinformatics, School of Medical Technology and Engineering, Fujian Medical University, Fuzhou, China; 3 Key Laboratory of Ministry of Education for Gastrointestinal Cancer, School of Basic Medical Sciences, Fujian Medical University, Fuzhou, China; 4 KIZ-CUHK Joint Laboratory of Bioresources and Molecular Research of Common Diseases, Kunming Institute of Zoology and The Chinese University of Hong Kong, Kunming, China; 5 CUHK Shenzhen Research Institute, Shenzhen, China; 6 Department of Psychiatry, The Chinese University of Hong Kong, Shatin, Hong Kong; 7 Margaret K.L. Cheung Research Centre for Management of Parkinsonism, The Chinese University of Hong Kong, Shatin, Hong Kong; 8 Brain and Mind Institute, The Chinese University of Hong Kong, Shatin, Hong Kong; 9 Hong Kong Branch of the Chinese Academy of Sciences (CAS) Center for Excellence in Animal Evolution and Genetics, The Chinese University of Hong Kong, Shatin, Hong Kong

**Keywords:** Medical genetics, Genomics, Psychiatric disorders, Diagnostic markers

Correction to: *Translational Psychiatry* 10.1038/s41398-021-01545-x, published online 13 August 2021

The original version of this article unfortunately contained a mistake. Recently, the authors found that there is an error in the equation 2.6. The original are has been corrected.

