# Supplementary material for: Analysis of genetic differences between psychiatric disorders: exploring pathways and cell types/tissues involved and ability to differentiate the disorders by polygenic scores
Source: Transl Psychiatry. 2021 Aug 13;11:426. doi: 10.1038/s41398-021-01545-x (PMC8363629; doi:10.1038/s41398-021-01545-x)
Supplement: Supplementary file 1 — Supplementary Text [file 41398_2021_1545_MOESM1_ESM.docx]

**Analysis of genetic differences between psychiatric disorders: exploring pathways and cell-types/tissues involved and ability to differentiate the disorders by polygenic scores**

Shitao RAO, Liangying YIN, Yong XIANG, Hon-Cheong SO

**Supplementary Text**

**Supplementary Methods**

**Quality control of SNPs and processing**

For further quality control, SNPs with a low imputation quality score (INFO R^2^ < 0.6) were excluded from further analysis. In addition, indels and duplicated SNPs were filtered. We then performed a harmonization step to keep the reference allele for signed test statistics consistent between each pair of GWAS datasets. Following that, the post–quality-control and harmonized summary statistics were utilized for investigating differential genetic variants for 26 comparisons of psychiatric disorders/traits (Table 3) using a statistical method presented below.

**Psychiatric traits/disorders included in the analysis**

As for the choice of disorders to compare, we intend to select pairs of disorders/traits which have some epidemiological associations and/or genetic overlap but are also distinct entities. For example, MDD is associated with a variety of other psychiatric disorders, such as SCZ^1^, anxiety disorders^2^, PTSD^3^, OCD^4^, alcohol, cannabis and other psychoactive substance use^5-8^, ASD^9^, ADHD^10^, ED^11^ etc., but each disorder also has its distinct clinical characteristics and aetiologies. As another example, neuroticism has been reported to be associated with other psychiatric disorders such as anxiety disorders, SCZ, MDD and AD ^12-14^. In addition, we included several other pairs of comparisons such as SCZ vs BPD^15^, ASD vs ADHD^16^ and AD vs cannabis use^17^, and they are related but also distinct psychiatric traits/diagnoses. We have included cannabis use as it is related to many psychiatric disorders^18^, and at the time of analysis, it is the only trait related to substance abuse (excluding alcohol dependence) with publicly available GWAS data and reasonably large sample sizes. Ideally a more well-characterized disorder or phenotype should be studied, which will be considered in our future work. Some other comparisons also included anxiety disorders and suicide attempts^19^ and psychotic experience with SCZ, BPD^20^ and MDD^21, 22^ due to possible clinical and/or genetic links between these traits/disorders.

*A note on the MDD GWAS sample*

For MDD which formed a major part of our comparisons, a recent GWAS meta-analysis was carried out based on 135,458 cases and 344,901 controls^23^. Excluding 23andMe data, the released GWAS summary statistics were generated from a sample set of 59,851 cases and 113,154 controls with a higher SNP-based heritability(7.8%, se 0.5%). The majority of MDD cases (45591 of 59851 cases, ~76.2%) in this sample were defined by clinical assessment or clinical records according to ICD/DSM criteria, although the UKBB sub-sample(14260/59851 cases) included some cases from self-reporting. While an updated study^24^ included a larger sample, the majority (excluding 23andMe) was composed of the broad depression phenotype in the UK Biobank dataset(127552/170756 cases); the sample also showed a lower SNP-based heritability(6.0%, se=0.3%)^24^. A recent study showed that genetic studies on depression defined by minimal or ‘broad’ phenotyping may not be specific to MDD itself^25^. Such studies might identify non-specific genetic factors linked to other psychiatric conditions; this may defy our purpose of finding differential genetic markers between related disorders/traits.

**Functional annotations of identified differential genetic markers**

The differential genetic variants identified were further explored for their biological functions using FUMA (<https://fuma.ctglab.nl/>)^26^. Following the definition by FUMA, independent significant SNPs were defined as those with *p*<5e-8 and independent from each other at the default *r*^2^ threshold (*r*^2^=0.6). For the definition of genomic *loci*, independent significant SNPs which are correlated with each other at *r*^2^ ≥ 0.1 are assigned to the same risk locus. Independent significant SNPs which lie within 250 kb are also merged into one genomic risk locus. All candidate SNPs in defined risk loci that are in LD (r^2^≥0.6) with the corresponding independent significant SNPs were selected for functional annotations, mainly including combined annotation-dependent depletion (CADD) scores^27^, chromatin states^28, 29^, ANNOVAR categories^30^ and RegulomeDB scores^31^.

**Gene mapping**

SNPs were mapped to genes in FUMA using three different strategies including mapping by position, expression quantitative trait loci (eQTL), and chro­matin interactions (CI). In brief, the positional method maps variants to genes based on their physical position, while the eQTL strategy maps SNPs to genes with which a significant (FDR<0.05) eQTL association exists. The third strategy (CI) maps SNPs to genes based on three-dimensional (3D) DNA-DNA interaction of the SNP and gene regions.

**Genome-wide gene-based association study (GWGAS) and tissue/cell-type enrichment analysis**

*P*-values from SNP-based analysis were utilized for GWGAS analysis in MAGMA ^32^. The program aggregates statistical significance of SNPs within a gene to output a gene-based statistic. Multiple testing was corrected by the false discovery rate (FDR) approach. In our gene-based and other analyses to follow, results with FDR<0.05 were considered significant.

The biological functions of GWGAS-significant genes were further investigated *via* tissue and cell-type expression enrichment analysis using MAGMA ^32^ and Linkage Disequilibrium SCore regression (LDSC) ^33^. In tissue enrichment analysis, MAGMA was used to test for enrichment based on over-representation of differentially expressed genes (DEGs) in each of 53 tissues in GTEx. We observed that brain regions were predominantly enriched in the above analysis; hence we focused subsequent analyses on the brain. Next we conducted an enrichment analysis *within* 13 brain regions using LDSC, based on GTEx data. This is a ‘competitive’ analysis restricted to the brain; the aim was to reveal enrichment within specific brain regions when compared to other regions.

Following that, all available single-cell expression datasets from human brain regions [Lateral Geniculate Nucleus (LGN), Middle Temporal Gyrus (MTG), hippocampus, cortex, prefrontal cortex, midbrain and temporal cortex] in FUMA were included for enrichment analysis to explore the specific types of contributing cells/neurons. A 2-step workflow was implemented for the enrichment analysis. The 1^st^ step was carried out to identify significantly enriched cell types, which were retained for the 2^nd^ step to determine independent signals within a dataset by stepwise conditional analysis (see also <https://fuma.ctglab.nl/tutorial#celltype>).

We also conducted pathway and gene-set enrichment analyses to explore whether these significantly associated genes were significantly enriched in biological predefined pathways or gene ontology (GO) sets based on the ConsensusPathDB database (CPDB, human) (<http://consensuspathdb.org/>)^34^.

**SNP-based heritability and genetic correlation with related traits**

SNP-based heritability (*h*^2^*_snp_*) of differential genetic associations was estimated by LDSC and SumHer ^35^. The former is the most widely used approach for estimating *h*^2^*_snp_*, and was employed as the primary estimation method here. We also performed additional analysis with SumHer, another program for *h*^2^*_snp_* estimation that allows more realistic heritability models. We also conducted ‘partitioned heritability’ analysis to identify which functional categories of genetic variants (e.g coding, promoter, histone marks, enhancers etc.) contribute the most to differentiation of the psychiatric disorders/traits ^36^.

In addition to shedding light on genetic architecture and relative importance of different functional categories, heritability explained is connected to the predictive power of genetic variants ^37^. In this regard, we also estimated the *maximum* ‘predictive ability’ (ability to differentiate the disorders in our case) that can be achieved if all variants on the GWAS panel are accounted for. Liability-scaled SNP-based heritability (*h*^2^*_snp_*) was calculated using LDSC, with sample and population prevalence as input. We estimated the ‘sample prevalence’ by the effective number of cases^38^ in the two datasets. We then followed the methodology described in ^37^ to compute different predictive indices and graphs. Briefly, we computed the AUC under ROC curve, proportion of cases explained by those at the top *k*% of predicted risk, variance of predicted risk and the absolute risk at different percentile. The graphs included ROC curve, predictiveness curve and the probability and cumulative density function of predicted risks. The analysis on differentiating ability was performed on comparisons of selected psychiatric disorders (SCZ, BPD, ED, ASD, ADHD, anxiety disorders, PTSD, OCD) and clinical symptoms (psychotic experience) for which differential diagnosis is considered more clinically relevant.

Genetic correlations (r_g_) between the differential genetic variations and 42 potentially related phenotypes were calculated using LDSC(<http://ldsc.broadinstitute.org/centers/>)^39^. Generally, r_g_ reflects how much the non-shared or unique genetic component of the 1^st^ disorder is genetically correlated with a specific trait, when compared to the 2^nd^ disorder in the pair. The rationale of this analysis is that when we consider two disorders as different, it is important to see whether they are associated with *different comorbid disorders/traits*. This distinction is important to help understand the different prognosis or aetiology of different disorders. For example, SCZ is generally associated with more prominent cognitive deficits than BPD. The above analysis may help to highlight such differences.

A set of 42 GWAS summary statistics were obtained from the LD-Hub^39^ and grouped into nine categories of traits including neurological diseases, personality traits, sleeping, cognitive, education, brain volume, psychiatric disorders, cardiometabolic traits and aging.

**Ability of polygenic risk scores (PRS) from existing GWAS data to differentiate disorders**

For selected traits for which differential diagnosis (DDx) are more clinically relevant, we performed another analysis to evaluate the ability of polygenic risk scores (PRS) from *existing* GWAS data to distinguish psychiatric disorders. The PRS was based on a case-control study of the corresponding disorders (disorder A as ‘case’ and disorder B as ‘control’). Note that unlike above, we are *not* focusing on the *maximum* predictive power achievable from all common variants.

An empirical Bayes approach has been proposed to recover the underlying effect sizes and could be used to forecast predictive ability of PRS, based on summary statistics alone^40^. The method has been verified in simulations and real data applications^40^. Eighteen subsets of genetic variants based on a series of *P*-value thresholds (10^−5^, 10^−4^, 5×10^−4^, 10^−3^, 5×10^−3^, 0.01, 0.03, 0.05, 0.1, 0.2, 0.3, 0.4, 0.5, 0.6, 0.7, 0.8, 0.9, and 1) were used to construct PRS.

**Additional details on the simulation model**

More specifically, we simulated standard normal variables *z_i_*~ *N*(0,1), and set mean effect size *µ* = $\sqrt{\frac{h^{2}}{N_{snp}}}$ . The actual effect size for SNP*_i_* was set at *β_i_* =*µ* * *z*_i_. The total liability *y* equals the sum of effects from each SNP plus a residual (*e*), i.e. $y=\sum_{i} \beta_{i}x_{i}+e$; the total variance of *y* was set to one. Following the liability threshold model, subjects with total liability exceeding a certain threshold [= $\Phi^{-1}\left( K \right)$, where *K* is the disease prevalence] are regarded as having the trait/disease. The non-shared genetic covariance between the two traits was set to 0.1.

**Supplementary Results**

Here we provide a more detailed description of the results.

**MDD against psychiatric disorders/outcomes**

In this part, we compared MDD with 12 different psychiatric disorders/outcomes, including SCZ, BPD, ED, ASD, ADHD, anxiety disorder, insomnia, alcohol dependence, ever used cannabis, SA, PTSD and OCD (Table 3). Totally 69 genomic risk loci were identified from the 12 pairs of comparisons(Table 3). Please refer to Table S2 to S13 for detailed results.

*MDD against SCZ*

Among the 12 pairs of comparisons, comparison of MDD and SCZ generate the largest number of genome-wide significant SNPs (2,312 SNPs, Table 3; sub-table 1 in Supplementary Table 3 (Table S2.1)) which belong to 37 genomic risk loci (Table S2.2). Although most of the candidate variants were located in intergenic and intronic regions (81% of variants) (Table S2.3), 65 SNPs were located in exons, including 32 nonsynonymous variants. Heritability enrichment analyses of 53 functional annotation categories indicated that the heritability of SNPs was not only enriched in intronic and conserved regions [Table S1.4; *P*<1.28E-04], but also in coding regions including transcription start site (TSS) (*P* = 1.78E-04). Besides, regulatory categories such as methylation and acetylation marks were found to be enriched among significant variants [*P* < 1.57E-04].

The three gene-mapping strategies (positional, eQTL and CI mapping) generated a set of 524 unique genes, 94 of which were implicated by all three methods (Table S2.5). Additionally, GWGAS analysis identified 953 significant genes (Table 3; Table S2.6). Taken together, 64 genes were implicated by all four strategies. Among them, *CACNA1C* was predicted to have a very high probability of loss of function mutation intolerance (pLI score=1; Table S2.5). Genes differentiating MDD and SCZ were mainly enriched in the cortex, the anterior cingulate cortex (BA24), and the frontal cortex (BA9) regions (Table S1.8;FDR<6.0E-04). Cell-type enrichment analysis suggested strong associations with several kinds of neurons in the cortex and prefrontal cortex (Table S2.9). Moreover, this analysis also identified associations with neurons in the midbrain, hippocampus, and lateral geniculate nucleus(LGN) regions(Table S2.9). Conditional analyses suggested neurons in the cortex, GABAergic neurons in the midbrain, and pyramidal neurons in the hippocampus as *independent* contributing neurons (after controlling for other cell types) (Table S2.10).

In gene-set enrichment analysis(GEA), the 953 GWGAS significant genes were enriched in a number of biological GO sets, including generation of neurons, regulation of nervous system development and central nervous system neuron differentiation [Table S2.11; FDR< 5.88E-03]. Other enriched pathways include neuronal system, alcoholism and brain-derived neurotrophic factor (BDNF) signalling pathway [Table S2.12; FDR< 1.86E-02]. In genetic correlation analysis, SCZ was defined as ‘case’ and MDD as (pseudo-)‘control’. Note that a positive genetic correlation indicates that the ‘case’ disorder is more positively associated with the studied trait genetically than the (pseudo-)‘control’ disorder, and vice versa. For example, we observed inverse genetic correlations(rg) with insomnia, neuroticism, coronary artery disease (CAD) and mean hippocampal volume, among others. This suggested that MDD has stronger positive genetic correlations with the above traits/disorders compared to SCZ. Findings of this type may shed light on different patterns of comorbidities, but may also be clinically informative. For instance, the significant inverse rg with CAD suggested that compared to SCZ patients, MDD patients may be more genetically predisposed to CAD.

*MDD against BPD, ED, ASD, ADHD, Anxiety disorder, Insomnia, Alcohol dependence* *and Cannabis use*

In these 8 pairs of comparisons, we identified 32 differential genomic loci (Table 3; detailed in Table S3-S10). The comparison between MDD and BPD revealed the largest number of significant genes based on GWGAS (174 genes; Table 3; Table S3.6). Here we just briefly highlight the comparisons of MDD with a few disorders (BPD, ADHD and anxiety disorders) which yielded the largest number of significant genes in GWGAS.

In the comparison between MDD and BPD, we found 4 significant risk loci, in which the strongest signal rs17751061 was found to be highly pathogenic likely influencing the function of *SUGP1* (Table S3.2, CADD = 35). Another nonsynonymous variant, rs17420378, was located in exon 8 of *STK4* (risk loci no. 4) with high predicted pathogenicity(Table S3.2, CADD=22.7). Besides, *STK4* was implicated by all four gene-mapping strategies(Table S3.5 and S3.6). The differential variants were enriched in brain regions including the cerebellum, cortex, and frontal cortex (BA9) [Table S3.7; FDR < 4.03E-02]. Enrichment analysis *within* brain regions suggested that the cortex and frontal cortex were the most enriched compared to others[Table S3.8; FDR<5.31E-04]. GEA revealed 174 GWGAS-significant genes, which were enriched in 17 gene ontology (GO) sets[Table S3.10; *P*<0.01], such as transitional metal ion binding and pre-mRNA binding. The most significantly enriched cell type was GABAergic neurons from LGN. Our pathway enrichment analysis indicated that differential genetic associations between MDD and BPD were involved in neural cell adhesion molecule (NCAM) signalling for neurite outgrowth, amphetamine addiction, serotonergic and glutamatergic synapse [Table S3.11;FDR = 4.88E-02 for all four pathways], among other pathways. Interestingly, the differential variants (BPD vs MDD) were found to have positive correlations with childhood IQ and a higher level of education [Table S3.12;FDR<3.26E-02], but negative correlations with insomnia and CAD [FDR< 4.92E-03].

In the comparison of MDD with ADHD, 167 significant differential genetic variants were identified which formed 5 genetic loci (Table S6.1 and S6.2). Four genes were mapped by all 3 gene-mapping strategies, including *KDM4A, SLC6A9, TMEM161B and CDH8* (Table S6.5 and S6.6). The most enriched cell types included GABAergic neurons in the midbrain and prefrontal cortex, as well as dopaminergic neurons in the midbrain (Table S6.9). Altogether 82 genes were significant in gene-based test, and pathway and GSEA shed light on pathways such as those related to DNA methylation (Table S6.10 and S6.11).

In the comparison between MDD and anxiety disorders, five genetic loci were found (Table 3), one of which involved the extended MHC (xMHC) region ^41^. The top mapped genes involved a set of genes in the xMHC region, and 3 other genes from other chromosomes (*LRFN5, PTPN1, FAM65C*) (Table 4). We note that due to the complex LD structure and high gene density in this region, it may be relatively difficult to identify the true casual gene/variant. GWGAS revealed 106 significant genes(Table S7.6). The most enriched tissues contributing to differential associations included the nucleus accumbens, frontal cortex and cerebellar hemisphere (Table S7.7); the most enriched cell types included GABAergic neurons from hippocampus, midbrain and temporal cortex, among others (Table S7.9).

**MDD against depression-related traits**

In this section, we tried to identify differential genetic variants from three pairs of comparisons between MDD and three depression-related phenotypes (probable recurrent severe depression, seen GP for anxiety/depression and longest period of feeling low/depressed). Ten risk loci were identified (Table 3, Table S14 to S16).

*MDD against depression defined in UKBB*

First we compared MDD (from PGC; majority clinically defined) against probable recurrent depression (severe)[ProbDep]. We identified 4 risk loci (Table 3;Table S13.2), including one in the xMHC region. Gene-based test revealed 110 significant genes. Tissue enrichment analysis highlighted the cerebellar hemisphere, nucleus accumbens and frontal cortex as the most enriched regions. Cell-type enrichment analysis suggested that the significant genes were associated with GABAergic, dopaminergic and other types of neurons in LGN, middle temporal gyrus (MTG), hippocampus, midbrain and cortex regions(Table S14.9). Pathway analysis mainly highlighted those related to DNA methylation and histone modification contributed by histone genes in the xMHC region; other top pathways included Rett syndrome causing genes and axon guidance pathway. GO sets enriched included regulation of long-term neuronal synaptic plasticity, central nervous system neuron development and differentiation(Table S14.11 and S14.12). Genetic correlation analysis showed that MDD-PGC was more positively genetically correlated with most other psychiatric disorders (e.g. SCZ, BPD, ASD, ADHD) as well as CAD when compared with ProbDep(Table S14.13). We then compared MDD against seen GP for nerves/anxiety/depression(GPDep). The significant variants mapped into 3 loci, 2 of which were also observed in the above analysis (including one in the xMHC region). Gene-based analysis revealed 72 genes; we observed an overlap of 69 genes with the previous analysis with ProbDep, although the latter analysis identified 110 significant genes. Other results of comparison between MDD and GPDep are shown in detail in Tables S15.

*MDD against duration of longest period of feeling low/depressed (top quintile as case)*

For this comparison, functional annotation of 133 candidate SNPs formed 3 genetic risk loci, among which the *GRIK2* gene was also mapped by the three gene-mapping methods (positional, eQTL, CI mapping; Table S16.5). The gene codes the Glutamate Ionotropic Receptor Kainate Type Subunit 2, suggesting glutamatergic transmission may be one factor with differential associations between susceptibility to depression and severity (as reflected by duration) of illness. Possibly due to limited sample size, tissue and cell-type enrichment analysis did not reveal significant results.

**Neuroticism against SCZ/MDD/Anxiety disorder/alcohol dependence**

In this part, five sets of GWAS summary statistics were employed which formed four pairs of comparisons (neuroticism against anxiety disorder, SCZ, MDD and alcohol dependence). The choice is based on relatively high association of neuroticism with these disorders ^12-14^. We identified 1,294 genomic risk loci from the four comparisons. For space limits, we highlight the results of neuroticism vs MDD only (Table S17). Please refer to Tables S18-S20 for detailed findings of other comparisons.

In the comparison of neuroticism against MDD, 20 risk loci were identified(Table S17.1 and S17.2). Functional annotations of 5,573 candidate SNPs in these loci highlighted a number of genes, among which *CRHR1, MAPT, WNT3* and *KANSL1* were mapped by all 3 gene-mapping methods and MAGMA (Table S17.5 and S17.6). They all belong to a risk locus on chr 17 but the exact causal gene(s) may require clarification in further studies. Tissue enrichment analysis observed enriched signals in most brain regions(Table S17.7); within-brain comparison showed that the cortex, frontal cortex, anterior cingulate cortex and nucleus accumbens were the most enriched [Table S17.8; FDR<1.40E-02]. Cell-type enrichment analysis of the GWGAS-significant genes suggested that they were mainly enriched in the LGN region (*P* (FDR within one dataset)<4.40E-02; Table S19.9). GO set enrichment analysis revealed that axon extension, CNS neuron differentiation and regulation of neuron death may be involved (Table S19.10).

**Psychotic experiences against SCZ/BPD/MDD**

Here we identified 10 and 2 genomic risk loci from comparison of psychotic experiences against SCZ and BPD respectively, but not from psychotic experiences against MDD (Table S21-S23).

*Psychotic experiences against SCZ*

In this comparison, functional annotation of 1,749 candidate SNPs revealed 10 genomic risk loci, covering 82 genes (Table S21.2). Altogether 68 genes were mapped by all three gene-mapping strategies, over half of which (35/68) was also indicated by GWGAS analysis (Table S21.5 and S21.6). The most implicated brain regions were the cortex, frontal cortex(BA9) and anterior cingulate cortex (Table S21.8). Cell-type enrichment analysis also observed significant signals in cortex and prefrontal cortex as the top two findings (Table S21.9). Enriched pathways or gene-sets included anterograde trans-synaptic signalling, synaptic vesicle exocytosis/ localization and synaptic adhesion-like molecules (Table S21.11 and S21.12).

*Psychotic experiences and BPD*

Functional annotation analysis highlighted several genes (*SUGP1, GATAD2A and CILP2*) harbouring SNPs with very high CADD scores (CADD score> 13.31, Table S22.2). The 3 gene-mapping strategies mapped variants to 10 genes(Table S22.5), all of which were also identified by GWGAS(Table S22.6). Tissue enrichment analysis suggested enrichment in the cortex, cerebellar hemisphere, frontal cortex, anterior cingulate cortex and cerebellum regions (FDR<2.99E-02; Table S22.7 and S22.8). Further analysis highlighted cocaine and amphetamine addiction, PTEN and EGF signalling as top pathways (Table S22.11).

*Psychotic experiences and MDD*

Although the comparison did not generate any significant differential variants, a few genes were highlighted via functional annotations of candidate SNPs (Table S23.2). All of the three gene-mapping strategies linked variants to 9 protein-coding genes (*TMEFF2, SLC30A9, BEND4, PRLR, EPM2A, FBXO30, SHPRH, GRM1, ANK3*; Table S23.5). Genetic correlation analysis suggested that MDD showed stronger rg with SCZ or BPD compared to psychotic experiences (Table S23.10).

**Other pairs of comparisons**

We also applied the proposed methods to the other four clinically relevant comparisons, including SCZ against BPD, ADHD against ASD, alcohol dependence against ever used cannabis and anxiety disorder against SA. We identified 3, 7, 2 and 1 genomic risk loci from each of the comparison respectively. Here we highlight the results from SCZ vs BPD and ADHD vs. ASD as examples (please refer to Tables S24-S27 for details).

*SCZ vs BPD*

Our analytic results based on GWAS summary data showed almost perfect genetic correlation with those obtained by comparing BPD and SCZ using *individual* genotype data ^42^ (r_g_ =1.054, se=0.025).

As for the actual results, we observed three significant loci in the comparison of SCZ and BPD (Table S24).

GWGAS highlighted 144 significant genes which were enriched for brain regions [Table S24.7; FDR<1.87E-02]. The frontal cortex and anterior cingulate cortex were the most enriched regions compared to other brain regions [Table S24.8; FDR< 2.55E-02]. Furthermore, cell-type enrichment analysis identified an enrichment signal in three different types of neurons in the midbrain region [Table S24.9; *P*<4.01E-03], which could withstand multiple testing correction within the corresponding dataset(FDR=3.34E-02). The enriched pathways included the inositol metabolism pathway and those related to cellular senescence [Table S24.11; FDR = 2.51E-02].

*ADHD vs ASD*

In the comparison between ADHD and ASD, 7 risk loci were found (Table S25.2). Seven genes (*KDM4A, ERI1, SOX7, PINX1, XKR6, MTMR9 and SEMA6D*; Table S25.5 and S25.6) were highlighted by all three gene-mapping methods and GWGAS (Table S25.5 and S25.6). We observed that ADHD may have stronger positive rg with CAD and insomnia compared to ASD; however, the reverse was observed for years of education, parental age at death and intra-cranial volume (Table S25.12).

**Overview of Supplementary Materials**

Supplementary Material 1: Detailed simulation results comparing analyses of individual-level genotype data ('real GWAS') and our analytic approach for differential genetic associations

Supplementary Materials 2 to 27: Full results (including further bioinformatics analysis) of differential genetic associations between different pairs of psychiatric disorders/traits. The order follows that listed in Table 3 of the main text.

Supplementary Materials 28 to 30: Differential genes identified by our proposed method, compared to those obtained by simple comparison of significant genes (FDR<0.01) from the 2 original GWAS. Supplementary Materials 28 to 30 present the full results for ASD vs ADHD, BPD vs MDD and SCZ vs BPD respectively.

**References**

1. Upthegrove R, Marwaha S, Birchwood M. Depression and Schizophrenia: Cause, Consequence, or Trans-diagnostic Issue? *Schizophrenia bulletin* 2017; **43**(2)**:** 240-244.

2. Hirschfeld RMA. The Comorbidity of Major Depression and Anxiety Disorders: Recognition and Management in Primary Care. *Prim Care Companion J Clin Psychiatry* 2001; **3**(6)**:** 244-254.

3. Bryant RA. Post-traumatic stress disorder: a state-of-the-art review of evidence and challenges. *World Psychiatry* 2019; **18**(3)**:** 259-269.

4. Goodwin GM. The overlap between anxiety, depression, and obsessive-compulsive disorder. *Dialogues Clin Neurosci* 2015; **17**(3)**:** 249-260.

5. McHugh RK, Weiss RD. Alcohol Use Disorder and Depressive Disorders. *Alcohol Res* 2019; **40**(1)**:** arcr.v40.41.01.

6. Lev-Ran S, Roerecke M, Le Foll B, George TP, McKenzie K, Rehm J. The association between cannabis use and depression: a systematic review and meta-analysis of longitudinal studies. *Psychological medicine* 2014; **44**(4)**:** 797-810.

7. Quello SB, Brady KT, Sonne SC. Mood disorders and substance use disorder: a complex comorbidity. *Sci Pract Perspect* 2005; **3**(1)**:** 13-21.

8. Gorfinkel LR, Stohl M, Hasin D. Association of Depression With Past-Month Cannabis Use Among US Adults Aged 20 to 59 Years, 2005 to 2016. *JAMA Network Open* 2020; **3**(8)**:** e2013802-e2013802.

9. Hollocks MJ, Lerh JW, Magiati I, Meiser-Stedman R, Brugha TS. Anxiety and depression in adults with autism spectrum disorder: a systematic review and meta-analysis. *Psychological medicine* 2019; **49**(4)**:** 559-572.

10. Daviss WB. A review of co-morbid depression in pediatric ADHD: Etiologies, phenomenology, and treatment. *Journal of child and adolescent psychopharmacology* 2008; **18**(6)**:** 565-571.

11. Mischoulon D, Eddy KT, Keshaviah A, Dinescu D, Ross SL, Kass AE *et al.* Depression and eating disorders: treatment and course. *Journal of affective disorders* 2011; **130**(3)**:** 470-477.

12. Xia J, He Q, Li Y, Xie D, Zhu S, Chen J *et al.* The relationship between neuroticism, major depressive disorder and comorbid disorders in Chinese women. *Journal of affective disorders* 2011; **135**(1-3)**:** 100-105.

13. van Os J, Jones P. Neuroticism as risk factor for schizophrenia. *Psychological medicine* 2001; **31:** 1129-1134.

14. Mosher Ruiz S, Oscar-Berman M, Kemppainen MI, Valmas MM, Sawyer KS. Associations Between Personality and Drinking Motives Among Abstinent Adult Alcoholic Men and Women. *Alcohol and Alcoholism* 2017; **52**(4)**:** 496-505.

15. Pearlson GD. Etiologic, Phenomenologic, and Endophenotypic Overlap of Schizophrenia and Bipolar Disorder. *Annual Review of Clinical Psychology* 2015; **11**(1)**:** 251-281.

16. Lau-Zhu A, Fritz A, McLoughlin G. Overlaps and distinctions between attention deficit/hyperactivity disorder and autism spectrum disorder in young adulthood: Systematic review and guiding framework for EEG-imaging research. *Neurosci Biobehav R* 2019; **96:** 93-115.

17. Yurasek AM, Aston ER, Metrik J. Co-use of Alcohol and Cannabis: A Review. *Current Addiction Reports* 2017; **4**(2)**:** 184-193.

18. Leweke FM, Koethe D. Cannabis and psychiatric disorders: it is not only addiction. *Addict Biol* 2008; **13**(2)**:** 264-275.

19. Nepon J, Belik S-L, Bolton J, Sareen J. The relationship between anxiety disorders and suicide attempts: findings from the National Epidemiologic Survey on Alcohol and Related Conditions. *Depress Anxiety* 2010; **27**(9)**:** 791-798.

20. Dunayevich E, Keck PE, Jr. Prevalence and description of psychotic features in bipolar mania. *Current psychiatry reports* 2000; **2**(4)**:** 286-290.

21. Owoeye O, Kingston T, Scully PJ, Baldwin P, Browne D, Kinsella A *et al.* Epidemiological and Clinical Characterization Following a First Psychotic Episode in Major Depressive Disorder: Comparisons With Schizophrenia and Bipolar I Disorder in the Cavan-Monaghan First Episode Psychosis Study (CAMFEPS). *Schizophrenia bulletin* 2013; **39**(4)**:** 756-765.

22. Legge SE, Jones HJ, Kendall KM, Pardiñas AF, Menzies G, Bracher-Smith M *et al.* Association of Genetic Liability to Psychotic Experiences With Neuropsychotic Disorders and Traits. *JAMA psychiatry* 2019; **76**(12)**:** 1256-1265.

23. Wray NR, Ripke S, Mattheisen M, Trzaskowski M, Byrne EM, Abdellaoui A *et al.* Genome-wide association analyses identify 44 risk variants and refine the genetic architecture of major depression. *Nature genetics* 2018; **50**(5)**:** 668-681.

24. Howard DM, Adams MJ, Clarke T-K, Hafferty JD, Gibson J, Shirali M *et al.* Genome-wide meta-analysis of depression identifies 102 independent variants and highlights the importance of the prefrontal brain regions. *Nature Neuroscience* 2019; **22**(3)**:** 343-352.

25. Cai N, Revez JAA, Adams MJ, Andlauer TF, Breen G, Byrne EM *et al.* Minimal phenotyping yields GWAS hits of reduced specificity for major depression. *BioRxiv* 2019**:** 440735.

26. Watanabe K, Taskesen E, van Bochoven A, Posthuma D. Functional mapping and annotation of genetic associations with FUMA. *Nat Commun* 2017; **8**(1)**:** 1826.

27. Kircher M, Witten DM, Jain P, O'Roak BJ, Cooper GM. A general framework for estimating the relative pathogenicity of human genetic variants. *Nature genetics* 2014; **46**(3)**:** 310-315.

28. Ernst J, Kellis M. ChromHMM: automating chromatin-state discovery and characterization. *Nature methods* 2012; **9**(3)**:** 215-216.

29. Kundaje A, Meuleman W, Ernst J, Bilenky M, Yen A, Heravi-Moussavi A *et al.* Integrative analysis of 111 reference human epigenomes. *Nature* 2015; **518**(7539)**:** 317-330.

30. Wang K, Li M, Hakonarson H. ANNOVAR: functional annotation of genetic variants from high-throughput sequencing data. *Nucleic acids research* 2010; **38**(16)**:** e164.

31. Boyle AP, Hong EL, Hariharan M, Cheng Y, Schaub MA, Kasowski M *et al.* Annotation of functional variation in personal genomes using RegulomeDB. *Genome research* 2012; **22**(9)**:** 1790-1797.

32. de Leeuw CA, Mooij JM, Heskes T, Posthuma D. MAGMA: Generalized Gene-Set Analysis of GWAS Data. *PLOS Computational Biology* 2015; **11**(4)**:** e1004219.

33. Bulik-Sullivan BK, Loh P-R, Finucane HK, Ripke S, Yang J, Patterson N *et al.* LD Score regression distinguishes confounding from polygenicity in genome-wide association studies. *Nature genetics* 2015; **47**(3)**:** 291-295.

34. Kamburov A, Stelzl U, Lehrach H, Herwig R. The ConsensusPathDB interaction database: 2013 update. *Nucleic Acids Research* 2012; **41**(D1)**:** D793-D800.

35. Speed D, Balding DJ. SumHer better estimates the SNP heritability of complex traits from summary statistics. *Nature genetics* 2019; **51**(2)**:** 277-284.

36. Finucane HK, Bulik-Sullivan B, Gusev A, Trynka G, Reshef Y, Loh P-R *et al.* Partitioning heritability by functional annotation using genome-wide association summary statistics. *Nature genetics* 2015; **47**(11)**:** 1228-1235.

37. So H-C, Sham PC. A unifying framework for evaluating the predictive power of genetic variants based on the level of heritability explained. *PLoS Genet* 2010; **6**(12)**:** e1001230-e1001230.

38. Boraska V, Jerončić A, Colonna V, Southam L, Nyholt DR, William Rayner N *et al.* Genome-wide meta-analysis of common variant differences between men and women. *Human molecular genetics* 2012; **21**(21)**:** 4805-4815.

39. Zheng J, Erzurumluoglu AM, Elsworth BL, Kemp JP, Howe L, Haycock PC *et al.* LD Hub: a centralized database and web interface to perform LD score regression that maximizes the potential of summary level GWAS data for SNP heritability and genetic correlation analysis. *Bioinformatics (Oxford, England)* 2017; **33**(2)**:** 272-279.

40. So HC, Sham PC. Improving polygenic risk prediction from summary statistics by an empirical Bayes approach. *Scientific reports* 2017; **7:** 41262.

41. Horton R, Wilming L, Rand V, Lovering RC, Bruford EA, Khodiyar VK *et al.* Gene map of the extended human MHC. *Nature Reviews Genetics* 2004; **5**(12)**:** 889-899.

42. BPD&SCZ-Working-Group-of-the-PGC. Genomic Dissection of Bipolar Disorder and Schizophrenia, Including 28 Subphenotypes. *Cell* 2018; **173**(7)**:** 1705-1715 e1716.
